# Cellular Partners of Tobamoviral Movement Proteins

**DOI:** 10.3390/ijms26010400

**Published:** 2025-01-05

**Authors:** Natalia M. Ershova, Kamila A. Kamarova, Ekaterina V. Sheshukova, Tatiana V. Komarova

**Affiliations:** 1 Vavilov Institute of General Genetics Russian Academy of Sciences, 119333 Moscow, Russia; ershova@vigg.ru (N.M.E.); kamila.kamarova@yandex.ru (K.A.K.); sheshukova@vigg.ru (E.V.S.); 2 Belozersky Institute of Physico-Chemical Biology, Lomonosov Moscow State University, 119991 Moscow, Russia

**Keywords:** movement protein, tobamovirus, tobacco mosaic virus, plasmodesmata, plant immunity, intercellular transport

## Abstract

The size of viral genomes is limited, thus the majority of encoded proteins possess multiple functions. The main function of tobamoviral movement protein (MP) is to perform plasmodesmata gating and mediate intercellular transport of the viral RNA. MP is a remarkable example of a protein that, in addition to the initially discovered and most obvious function, carries out numerous activities that are important both for the manifestation of its key function and for successful and productive infection in general. Briefly, MP binds the viral genome, delivers it to the plasmodesmata (PD) and mediates its intercellular transfer. To implement the transport function, MP interacts with diverse cellular factors. Each of these cellular proteins has its own function, which could be different under normal conditions and upon viral infection. Here, we summarize the data available at present on the plethora of cellular factors that were identified as tobamoviral MP partners and analyze the role of these interactions in infection development.

## 1. Introduction

Plants have evolved a complex, highly regulated, and multi-layered defense system to counteract viral pathogens. A virus entering the cell and initiating infection activates so-called dominant defense responses, such as innate immunity, RNA interference (RNAi), and a number of other reactions [1,2,3,4,5,6,7]. Pathogen-associated molecular patterns (PAMPs) induce a plant response designated as PAMP-triggered immunity (PTI), which is activated when pattern-recognition receptors (PRRs) on the cell surface perceive PAMPs [8,9]. To overcome the first line of defense, pathogens produce effectors that allow the pathogen to evade or block PTI, resulting in the establishment of cellular effector-triggered susceptibility (ETS). Counteracting this virulence strategy, the plant cell activates receptor-like proteins encoded by genes of resistance (R) that recognize effectors—avirulence (Avr) factors—produced by the pathogen. R factors directly or indirectly interact with pathogens’ Avr factors mediating effector-triggered immunity (ETI) to the pathogen [7,10]. These co-evolving pathogen virulence strategies and plant resistance mechanisms illustrate an evolutionary arms race between the pathogen and the host that is integrated into the zigzag model of plant innate immunity and is defined by the formula [PTI-ETS+ETI]. ETI, and in some cases PTI, may culminate in a hypersensitive response (HR) and resistance to a pathogen [8]. For a long time, the zigzag model of plant immunity excluded the plant–virus relationship because viruses, being intracellular parasites, were believed not to possess signatures recognized as PAMPs and thus to be unable to induce PTI [5]. The main paradigms of immune antiviral responses included HR and systemic acquired resistance [11,12]. Over a decade ago, the concept describing plant immunity was supplemented with new terms—viral effectors (double-stranded RNA (dsRNA) and viral proteins), viral PTI, and viral ETI—which are now integrated into the modern model of plant immune responses [5].

Innate immunity and RNAi are the main defense mechanisms against viruses in plants, acting at the initial stages of infection [5,7,13,14,15]. In addition, other types of virus-induced defense responses have been described, including translation repression, ubiquitinylation- and autophagy-mediated protein degradation, and nonsense-mediated decay of viral RNA [1,2,3,4,5,6,7]. Last decade, several novel dominant resistance genes were identified, which encode atypical dominant viral resistance proteins (ADVRPs) [15]. ADVRPs do not have structural similarity with each other and with the known products of R genes; moreover, the list of these proteins seems not to be exhaustive and novel members of this group are being discovered.

**Figure 1:**
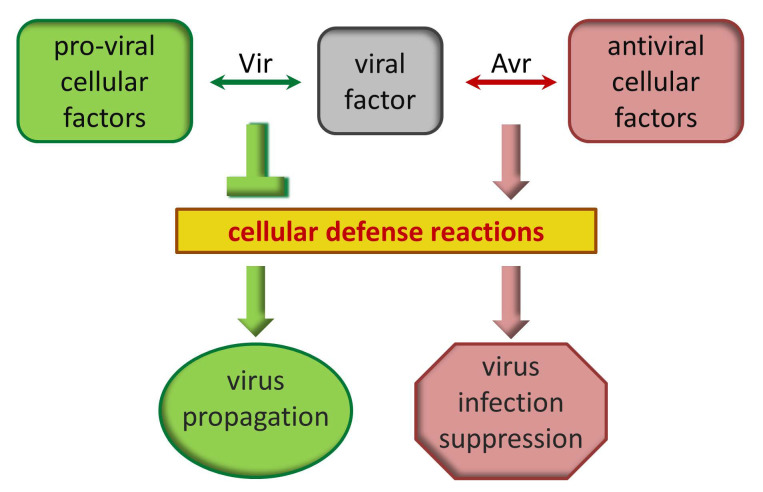
Multifunctional viral proteins interact with cellular pro- and antiviral factors. Depending on the cellular partner, the viral component acts as virulence (Vir) or avirulence (Avr) factor, thus suppressing or activating host defense response resulting in facilitation or suppression of viral infection.

Studies of dominant R genes have revealed a number of antiviral proteins that limit virus propagation by directly interacting with viral proteins and inhibiting their functions. Similar to innate immunity, the RNAi system also recognizes dsRNA molecules of viral origin, carries out signal amplification, and establishes resistance to viral infection in uninfected tissues through intercellular and systemic trafficking of viral-specific short interfering RNA (siRNA) duplexes [16,17]. In addition to RNAi-based plant defense mechanism, there are a plethora of cellular factors that participate in the abovementioned plant responses or other reactions during host–virus interactions. Moreover, their functions could differ in normal conditions and upon stress. Such cellular factors are designated as proteins with pro- or antiviral activity [18,19]. Pro-viral host factors act in concert with viral factors and are essential for the key processes of the viral reproduction cycle and successful infection development. Antiviral host factors, on the contrary, affect particular viral components—nucleic acids or proteins—interfering with their functioning directly or indirectly, and therefore mediating suppression of the infection. Thus, each viral component may play a dual role in the context of plant defense: it could be simultaneously an Avr factor for the antiviral proteins, interaction with which results in suppression of infection, and a virulence (Vir) factor when interacting with pro-viral proteins that facilitate infection (Figure 1).

Tobacco mosaic virus (TMV) is a type member of the genus *Tobamovirus* and one of the best-studied plant viruses. Its genome consists of a single-stranded positive RNA that encodes two components of replicase complex, 126-kDa and 183-kDa proteins, the movement protein (MP), and the coat protein (CP) [20]. In addition, TMV genome contains an open reading frame (ORF) for a putative 54-kDa protein with an undefined role and an ORF encoding 4.8-kDa protein that affects symptomology [21,22]. ORFs for replicase components, MP and CP, are present in all tobamovirus genomes, while an ORF for 4.8-kDa protein is characteristic only for some tobamoviruses [20].

Replicase, MP, and CP can act as Avr determinants, meeting in the cell with their R factors. *Nicotiana glutinosa N* gene was the first identified R gene that mediates plant resistance to tobamoviral infection [23,24]. *Nicotiana sylvestris* protein encoded by *N′* gene recognizes tobamoviral CP, which results in HR induction [25,26]. N protein indirectly interacts with the helicase domain of the TMV 126 kDa replicase component, inducing plant resistance [27]. At the same time, tobamovirus replicase proteins possess anti-silencing activity, i.e., are viral suppressors of RNA silencing. Replicase components 126 kDa of TMV and 125 kDa of oilseed rape mosaic virus (ORMV) inhibit HEN1 (HUA ENHANCER1) methyltransferase activity, thus hampering methylation of siRNA and suppressing RNAi [28,29,30,31,32]. Turnip vein-clearing virus (TVCV) 122 kDa protein binds dsRNA, siRNA and micro RNA (miRNA), and thus blocks RISC complex activation [33]. Tobamovirus MPs are able to suppress PTI induced by viral dsRNA [34]. Based on the multifunctionality of tobamovirus replicase and MPs, the following model of viral infection strategy was proposed. It describes these proteins as Vir effectors. TMV, ORMV, and TVCV MPs perform their basic function binding viral RNA and transporting it from cell to cell, on the one hand, and on the other hand, they act as Vir factors, interacting at the leading edge of infection with components mediating dsRNA-induced PTI, therefore suppressing this branch of immune response. Proteins of replicase complex, in addition to their main function of viral RNA replication, act as Vir factors inside the focus of infection where they suppress dsRNA-induced antiviral RNAi due to their anti-silencing activity [34].

Tobamovirus MP binds viral genome, mediates its intracellular targeting to plasmodesmata (PD), and performs PD gating to further facilitate viral RNA movement to the neighboring cells [35,36]. At every stage of infection, MP interacts with multiple cellular partners. However, MP’s ability to directly bind PD components, thus inducing an increase in PD permeability, is not documented [35]. It is believed that MP initially interacts with PD-regulating proteins which then affect PD size exclusion limit. In addition to PD localization, TMV MP has been shown to be associated with endoplasmic reticulum (ER), actin filaments, and microtubules [37,38]. It is suggested that MP re-localizes from PD to microtubules behind the leading edge because its transport function is not essential at the late stages of infection and thus MP should be “withdrawn”. MP proteasomal degradation could serve the same purpose—regulating the amount of MP during infection development [39,40]. These mechanisms could represent an example of the cellular systems exploitation beneficial for virus.

To perform its functions, TMV MP interacts with various cellular components, including cytoskeletal proteins, actin and myosins, membrane components and chaperones of the endoplasmic reticulum, pectin methylesterase, kinases, ER and plasma membrane proteins (synaptotagmins, remorins, calreticulin, proteins containing ankyrin domain, etc.) (Table 1). Moreover, MPs of closely related to TMV tobamoviruses were reported to interact with Rubisco small subunit [41] and nuclear components [42,43].

The study of MP cellular interactors widens our knowledge of the plant–virus pathosystem functioning. The global aim of such research is to develop the instruments and approaches, based on the principles and mechanisms underlying these interactions, that make it possible to protect plants from viral diseases and produce tolerant or resistant variants of crop plants.

Here, we analyze data available at present on cellular factors identified as interacting partners of tobamoviral MPs, in particular TMV MP, pay special attention to the approaches used for their identification, and discuss the role of these cellular proteins in the development of infection and their significance for plant–virus interactions.

## 2. Cytoskeleton

The cytoskeleton, composed of microtubules, actin filaments, and intermediate filaments, plays a pivotal role in maintaining cellular structure, intracellular transport, and signal transduction [79]. Understanding how TMV MP interacts with the host cytoskeleton and cytoskeleton-related proteins is essential for comprehending the mechanisms underlying TMV infection and spread. TMV MP has been shown to interact with the cytoskeleton to facilitate viral movement within the host cells and between cells. The TMV MP interacts with the cytoskeleton by binding to microtubules and actin filaments. These interactions are critical for the intracellular transport of viral RNA to the PD.

### 2.1. Microtubules and Tubulin

Microtubules are cylindrical structures consisting of tubulin and playing a key role in intracellular transport and cell division [80]. Early research on the intracellular movement of plant viruses mainly examined the relationship between the TMV MP and microtubules. The TMV MP was reported to bind tubulin in vitro [44] and microtubules in vitro and in vivo [45,81,82]. Tubulin-like motifs similar to those mediating lateral contacts between microtubule profilaments are believed to be responsible for MP/microtubule interaction [83]. It was hypothesized that microtubules’ dynamics at the leading edge of infection might drive the intracellular transport of MP or MP/RNA complex to PD [46,47]. Supporting this hypothesis, tobacco mutants with defective microtubule dynamics show reduced susceptibility to TMV infection [50]. Noteworthy, some reports challenge the importance of the MP/microtubule interaction for TMV cell-to-cell movement. For example, disrupting microtubule structure or tubulin polymerization with drugs or inducing tubulin gene silencing did not affect TMV intercellular movement, and TMV MP mutants with lower affinity for microtubules did not impede virus spread [48]. Wright et al. [84] demonstrated that microtubule polymerization inhibitors colchicine and oryzalin did not prevent MP intracellular movement to PD. However, the experimental setup based on pharmacological inhibitors was contested and microtubule-independent MP intracellular transport was brought into a question [85]. Nevertheless, it is commonly accepted that MP/microtubule interactions play an important role in TMV local spread and virus factories formation but are possibly not essential for mediating MP trafficking to PD [86]. During the late stages of infection, TMV MP associates with microtubules but remains stationary, suggesting a possible degradation pathway, even though the microtubule-bound MP is not ubiquitinylated [82]. Moreover, at least two proteins that are able to bind both MP and microtubules were identified: movement protein binding 2C (MPB2C) [54] and microtubule end-binding protein (EBP1) [56], which are discussed below.

### 2.2. Microtubule-Associated Protein MPB2C

*Nicotiana tabacum* MPB2C was isolated and identified as an MP partner using a yeast SOS recruitment system (SRS) which is suitable for membrane-associated protein analysis [54]. To clarify the role of MPB2C/MP interaction for TMV infection development, both the effect of *MPB2C* gene overexpression [54] and knockdown [55] were studied. *MPB2C* transient expression negatively affects MP intercellular transport in *N. benthamiana*, decreasing the efficiency of MP transport into neighboring cells two-fold. In addition, MP intracellular localization changed: the protein re-distributed along microtubules while the amount of PD-localized MP decreased [54]. Surprisingly, downregulation of *MPB2C* expression via virus-induced gene silencing (VIGS) did not affect either MP intercellular transport or TMV infection spread. However, reduced *MPB2C* expression led to a distinct change in the intracellular distribution of MP: it was almost completely re-distributed to PD, losing co-localization with microtubules [55]. According to the authors’ model, MPB2C “retains” MP on microtubules, preventing its targeting to PD that leads to the decreased efficiency of the intercellular transport. It could also be suggested that MP is targeted to 26S proteasome for degradation by MPB2C as occurs with the transcription factor KNOTTED1 [39,87]. Later, Ruggenthaler et al. (2009) [88] obtained results supporting the suggested model. The study of *Arabidopsis thaliana* MPB2C homolog demonstrated that transgenic plants overexpressing a cassette encoding GFP-AtMPB2C fusion protein are resistant to tobamoviral infection (oilseed rape mosaic virus) [88]. 

Thus, artificial MPB2C upregulation results in plant resistance to tobamovirus infection. However, the absence of an infection-stimulating effect in plants with *MPB2C* knockdown does not allow us to unambiguously classify MPB2C as an antiviral or pro-viral factor. Upon transient expression or in transgenic plants, the MPB2C level is evenly increased throughout the leaf, while in natural conditions it might be different. For instance, at the leading edge of infection the MPB2C level is low, and thus does not have a negative effect on transport, while inside the focus of infection MPB2C might be upregulated, resulting in the decreased efficiency of intercellular transport and MP relocalization to microtubules characteristic of the later stages of infection [45]. However, in the abovementioned studies, the TMV RNA levels were not assessed, and there are therefore no data on the MPB2C effect on replication and viral RNA intracellular transport. Moreover, it could not be excluded that MP redistribution could affect both. Thus, the role of MPB2C/MP interactions seems to be understudied and needs to be further elucidated.

### 2.3. Microtubule End-Binding Protein (EBP1)

EBP1 is another microtubule-binding protein that was demonstrated to co-localize and bind TMV MP in vitro and in vivo. Transient overexpression of a gene encoding AtEBP1:GFP fusion protein led to the decreased efficiency of TMV intercellular transport [56]. In leaves with elevated *AtEB1:GFP* expression, MP:RFP is localized to microtubules at the leading front of infection; thus, EBP1 likely tethers MP to microtubules, sequestering it. It could be suggested that EBP1, similar to MPB2C, plays a regulatory role in MP functioning.

### 2.4. Actin

Actin filaments are essential for maintaining cell shape and enabling intracellular transport. TMV MP interacts with actin filaments, as demonstrated by studies showing MP co-localizing with actin in infected cells [44,89]. This interaction disrupts the normal organization of actin filaments, often leading to the formation of dense actin networks that aid the transport of viral RNA. The rearrangement of actin filaments by MP is crucial for the efficient movement of TMV within the plant [53]. The important role of actin filaments was also shown for turnip vein clearing virus (TVCV) MP. In contrast to TMV MP, MP^TVCV^ was demonstrated not to be associated with microtubules. Instead of this, MP^TVCV^ enters the nucleus and interacts with chromatin-associated F-actin filaments there. Within the nucleus, MP^TVCV^ did not co-localize with nucleoli or Cajal bodies but did co-localize with histone H2B [42]. These findings suggest that MP^TVCV^ might directly influence nuclear actin dynamics to modify gene expression, potentially increasing the TVCV virulence. Such interactions were shown to be necessary for efficient TVCV local spread and systemic infection in *N. benthamiana* and *A. thaliana* [42,43].

### 2.5. Motor Proteins

Motor proteins such as kinesin, dynein, and myosin are responsible for the transport of cargo along microtubules and actin filaments. TMV MP interacts with these motor proteins to facilitate the movement of viral components. Different myosins are involved in various stages of TMV movement. Myosins XI-2 and XI-K are crucial for TMV intracellular movement, aiding ER-associated transport [51,52]. In contrast, myosins VIII-1, VIII-2, and VIII-B assist in the movement by specifically targeting TMV MP to PD [51].

In summary, the interaction of TMV MP with the cytoskeleton is a crucial aspect of viral movement and pathogenicity. By binding to microtubules, actin filaments, and motor proteins, MP facilitates the transport of viral RNA to the PD and its subsequent passage into neighboring cells. Moreover, MP^TVCV^ intrudes the nucleus, where it associates with F-actin and histone H2B, possibly affecting nuclear dynamics and gene expression. These interactions not only promote the efficient spread of TMV within the host plant but also illustrate the complex strategies viruses employ to hijack host cellular machinery and aiming at the suppression of the plant defense responses or the activation of pro-viral factors expression. Therefore, cytoskeleton components could unquestionably be regarded as cellular factors essential for viral infection, and thus pro-viral. However, some components, for example MPB2C or EBP1, are likely not the stimulators of the viral infection but rather the regulators that mediate the switch between different stages of the infection process.

## 3. Membrane Contact Sites

Membrane contact sites (MCS) are the regions where membranes of different organelles or cellular compartments physically interact but do not fuse [90]. These sites were first characterized due to their critical role in non-vesicular transport of small molecules and lipids, and intracellular calcium ion exchange [91,92,93]. MCS harbor a specific set of proteins and lipids that are critical for membrane flexibility, serving as a site for regulatory protein complex assembly [94]. Specialized ER–plasma membrane (PM) MCSs are believed to be the structural components of PD: the desmotubule, which continuously passes through the PD channel, is closely adjacent to the PM (~10 nm) and is tethered to the PM-lined channel by spoke-like proteins, the functions and identity of which are the subject of many studies [95,96,97]. In the context of viral infection, PD-associated MCS between ER and PM are regarded as structures exploited by viruses for both movement and replication [98]. Several proteins participating in maintaining ER–PM MCSs have been identified [99]. Among them are the proteins that were shown to interact with tobamoviral MPs: synaptotagmins, reticulons, and remorins, which are discussed below.

### 3.1. Synaptotagmin A (SYTA, SYT1)

*A. thaliana* synaptotagmin A (SYTA, or SYT1) is a member of a large family of synaptotagmins (SYTs), homologs of which are present in all eukaryotes. SYT1 regulates endocytosis and is involved in the recycling of endosomal vesicles during viral infection. It participates in the formation of ER–PM contact sites, being one of the tethering proteins [96,100]. Ishikawa et al. (2020) [101] have demonstrated that two other SYTs, SYT5 and SYT7, interact with SYT1 and are essential for ER–PM contact formation. The majority of SYT-containing MCS are localized around PD [59,101]. SYT1 was shown to be a cellular factor interacting with TMV MP and mediating its PD accumulation. Inhibition of TMV MP intercellular movement was demonstrated in the *A. thaliana* transgenic line with downregulated *SYT1* expression or in *N. benthamiana* plants with transient expression of a dominant-negative SYTA mutant [57]. Later, it was shown that TVCV and TMV MPs interact directly with SYT1 and SYT1 is essential for targeting MP to PD [58]. Moreover, Yuan et al. (2018) [102] reported that first 50 N-terminal amino acid residues of MP, recognized as its plasmodesmata-localization signal (PLS) [103], are necessary and sufficient for interaction with SYT1 and for MP localization at PD. However, in 2020 Dr Yuan’s group identified two additional MP domains containing amino acid residues from 61 to 80 and from 147 to 170 that are responsible for MP PD localization together with the major PLS signal [60]. From these two domains, the first one (61–80) was shown to interact with SYT1. Ishikawa et al. (2020) reported the important role of three synaptotagmins, SYT1, SYT5, and SYT7, in the anchoring ER membrane to PM, especially around PD, and their significance for viral cell-to-cell movement. In *syt1/syt5/syt7* triple mutant arabidopsis Youcai mosaic virus (YoMV) MP fused to GFP failed to localize to PD and its intercellular transport was drastically reduced compared to the wild-type plants. Interestingly, *syt1*, *syt5*, or *syt7* single mutants supported similar efficiency of YoMV infection development as wild-type plants, while double or triple mutants appeared to be tolerant to YoMV [101]. SYT1 is not essential for the intercellular transport of conventionally secreted proteins [59,101,104] but is indispensable for tobamoviral MPs delivery to PD.

Therefore, according to existing data, SYT1 is exploited by tobamoviruses for MP targeting to PD and is essential for productive TMV and TVCV infection.

### 3.2. Remorins

Plant remorins, which are characterized by localization in nanodomains, are found in the PM and PD. The remorin family is represented by proteins anchored in the PM of plant cells and regulating the aperture and functionality of plasmodesmata [105,106,107]. They play an important role in plant responses to various stress factors, including viral infection [62,108,109]. *N. benthamiana* remorins belonging to the group 1 are palmitoylated and localized to the PM [61]. Of the four selected for the analysis NbREMs of the group 1 (NbREM1.1, 1.3, 1.5, 1.8), only remorin NbREM1.5 was demonstrated to affect TMV:GFP local spread: *NbREM1.5* overexpression suppressed TMV:GFP transport, while its VIGS-mediated downregulation stimulated TMV:GFP intercellular movement [61]. NbREM1.5 mutant variants lacking palmitate accumulated at lower levels and did not affect MP-mediated cell-to-cell transport of the reporter molecule; therefore, this modification appeared to be essential for NbREM1.5 correct functioning. In addition to direct interaction with TMV MP, NbREM1.5 stimulated PD callose deposition. Thus, the authors suggest that there are two mechanisms underlying NbREM1.5’s ability to restrict TMV intercellular transport: negative regulation of PD SEL by enhancing callose deposition and interfering with MP functioning by interacting with this protein [61]. Although the effect of NbREM1.2 on TMV transport was not reported, its homolog NtREM1.2 from *N. tabacum* was characterized by Sasaki et al. (2018) [62]. NtREM1.2, in contrast to NbREM1.5, was demonstrated to stimulate local spread of closely related to TMV tomato mosaic virus (ToMV) when expressed in *N. benthamiana* plants. Moreover, NtREM1.2 was shown to directly interact with ToMV MP in BiFC assay.

Thus, the remorin story is far from clear. Different members of this protein family have an opposite effect on viral transport. For instance, *Solanum tuberosum* StREM1.3 hampers TMV MP- and potato virus X (PVX) TGB1-mediated PD gating activity [108,109] but stimulates turnip mosaic virus (TuMV) and potato virus A (PVA) local spread [110].

To summarize, remorins, being membrane-anchored proteins localized to microdomains of PM and regulating PD permeability, affecting PD callose deposition, definitely play an important role in viral, and in particular tobamoviral, cell-to-cell movement. However, due to the differences in the effects, they could not be unequivocally categorized as pro- or antiviral cellular factors.

### 3.3. Reticulons and Reticulon-like Proteins

Reticulons (RTNs) are integral membrane proteins that could form dimers or oligomers and thus create local tensions inducing membrane bending and shaping. The members of this family have been found in all eukaryotes studied so far [111]. RTNs are predominantly localized to ER and play a central role in mediating the correct morphology of ER membranes. Plant members of the reticulon protein family, RTNLB3 and RTNLB6, have been found in the PD proteome [112]. They contain reticulon homology domain (RHD) that includes two hydrophobic regions spanning the membrane in such a way that RTNLB molecule becomes W-shaped with a C- and an N-termini facing the cytosol [113]. RTNLBs are believed to contribute to desmotubule formation by generating a unique membrane curvature in primary PD due to their membrane constriction properties [114]. However, in mature tissues, RTNLB3 and RTNLB6 also remain associated with PD desmotubule and co-localize with TMV MP [114]. Among proteins that were shown to interact with RTNLB3 and RTNLB6, there are SYT1 and two remorins—REM1.2 and REM1.3 [113]. Thus, it could be speculated that TMV MP, SYT1, RTNLBs, and REMs might operate in complex facilitating viral cell-to-cell transport. Recently, RTNLB3 and RTNLB6 were found to directly interact with the MPs of TMV and several other viruses [63]. Co-expression of RTNLB3 with cucumber mosaic virus (CMV) movement protein 3a resulted in decreased intercellular movement of the reporter protein (GFP), indicating that RTNLB3/3a interaction interferes with 3a transport function. The authors suggest that RTNLBs might be exploited by viruses for a variety of purposes, including modification of cell membrane architecture and composition to facilitate viral replication, targeting to and movement though PD, and direct or indirect modification of PD [63].

The question of the pro- or antiviral role of reticulons remains unresolved and is to be further elucidated.

## 4. Ankyrin Repeat-Containing Protein ANK

Three cellular factors that interact with PVX TGB2 protein were identified in *N. tabacum* cv. Samsun NN and shown to participate in PVX intercellular transport via a callose-dependent mechanism. They were designated as TGB12K-interacting proteins (TIPs) 1–3. TIPs appeared to be ankyrin repeat-containing proteins [115]. Later, TMV MP was also demonstrated to interact with ankyrin repeat-containing protein ANK, which was identified from another tobacco cultivar—*N. tabacum* cv. Turk—and was shown to have the highest degree of similarity with TIP1 [64]. In general, ankyrin repeat-containing proteins have various functions both during development, in particular chloroplast biogenesis [116,117,118], and in response to external stimuli, stress, and pathogens [119,120,121,122]. Noteworthy, ankyrin repeat-containing proteins are often transmembrane and ankyrin domains are responsible for protein–protein interactions [123,124]. It was demonstrated that transgenic *N. tabacum* plants with elevated ANK levels supported more effective intercellular transport of MP-YFP fusion protein or TMV-dsRed viral vector than plants with *ANK* expression suppressed by RNAi. Co-expression of ANK and TMV MP induced PD callose reduction [64]. The question of how ANK proteins affect PD callose is still to be elucidated: Fridborg et al. [115] suggested that ANK interacts with a callose-degrading enzyme 1,3-glucanase based on the results obtained using the yeast two-hybrid (Y2H) system and the far-Western approach, while Ueki et al. [64] argue against this hypothesis, pointing out different topology of these proteins and suggesting ANK potential interaction with one of the components of callose synthase. However, regardless of the underlying mechanism, ANK/MP interaction facilitates viral intercellular transport. Thus, ANK is a cellular protein exploited by TMV for effective cell-to-cell movement. This provides an opportunity to claim that ANK is a pro-viral factor.

## 5. Chaperones and Chaperone-like Proteins

Tobamoviral infection induces reformatting of the cellular ER membrane system exploiting it for the formation of so-called viral factories where replication occurs [125]. In addition, massive production of the foreign for the cell viral proteins results in ER stress and overload. Consequently, the cell responds with the activation of the protein quality control system and more intensive targeting of viral proteins to degradation via the ubiquitin-dependent pathway to 26S proteasome. Moreover, it was demonstrated that during N-gene-mediated HR, numerous ER resident chaperones are upregulated [126,127]. They are believed to play a significant role in the accumulation of membrane or secreted proteins involved in plant innate immunity. In addition, the virus might activate ubiquitinylation and degradation of some cellular antiviral proteins [3]. And, finally, the virus is likely to exploit proteasomal and aggresomal pathways of protein degradation to regulate the amount of its own proteins, in particular MP, at different stages of infection [39,128]. Thus, the ER quality control system and especially chaperones play a significant role during viral infection. Below, several ER proteins that were demonstrated to directly interact with MP are discussed.

### 5.1. MP Interacting Protein 1 (NtMPIP1)

NtMPIP1 is a member of the type I DnaJ chaperone family [65]. This protein was demonstrated to directly interact with TMV MP in the Y2H system as well as in a blot overlay assay. Moreover, NtMPIP1 appeared to be a partner of another protein, NTH201, a class II KNOTTED1-like protein, that was earlier demonstrated to facilitate TMV intercellular spread and to co-localize, but not being in contact with MP [129]. Shimuzu et al. (2009) [65] showed that MP/NtMPIP1/NTH201 interact in the yeast three-hybrid system. Thus, NtMPIP1 likely serves as a connecting link between MP and NTH201. Downregulation of either *NTH201* [129] or *NtMPIP1* [65] expression using VIGS led to the suppression of TMV intercellular spread and its RNA accumulation. Therefore, despite the completely different functions and nature of these proteins (NTH201 represents transcription factor-like proteins while NtMPIP1 belongs to the DnaJ chaperone family), they both could be regarded as pro-viral cellular factors acting ensemble.

### 5.2. CELL-DIVISION CYCLE Protein 48 (CDC48)

CDC48 is a chaperone that plays a significant role in plant immunity, being one of the key players of the ER protein quality control system [127]. It functions in ER membrane maintenance upon ER stress and participates in protein retrotranslocation from ER to the cytoplasm, targeting them for degradation. Moreover, CDC48 was found among ER-resident chaperons participating in N-protein-mediated HR to TMV infection [126]. CDC48 was demonstrated to be upregulated upon tobamoviral infection and to directly interact with TMV MP in vitro and in vivo [40]. According to Niehl et al., CDC48-mediated translocation of MP from the ER-associated inclusions (that contain viral factories) at the late stage of infection could be a means by which the virus targets excessive amounts of MP for degradation to the ubiquitin–proteasome system (UPS) and/or “switches” from active intercellular spread to replication and virion production [40,128]. It cannot be ruled out that the degradation of viral proteins during infection development may represent their normal physiological turnover [130]. Small amounts of MP are sufficient for binding to viral RNA for its transport to PD and through PD. Thus, accumulation of functional MP is essential only at the leading edge of infection [36,131] where it performs PD gating. At the late stages of infection, i.e., behind the leading edge and inside the infection focus, MP was still detected in the PD, however it lost the ability to mediate intercellular transport [132].

Although CDC48 overexpression ahead of infection leads to suppression of the virus intercellular spread, this factor could not be regarded as antiviral. CDC48 might perform as a mediator that is exploited by the virus for MP relocalization to microtubules in order to prevent virus cell-to-cell movement and enhance progeny production. Thus, CDC48/MP interaction allows for maintaining such MP level that is essential for the particular stage of infection.

### 5.3. Calreticulins (CRTs)

CRTs are ER-resident chaperons responsible for Ca^2+^ homeostasis and protein folding [133]. Furthermore, there are studies reporting that some plant CRTs possess specialized functions, e.g., *A. thaliana* CRT3 is involved in innate immune response participating in PAMP recognition [134] and is responsible for retaining defective forms of brassinosteroid receptor BRI1 in the ER [135]. Moreover, there is evidence that plant CRTs are localized to PD [133,134,136,137] and are involved in PD permeability regulation. *N. benthamiana* NbCRT2 and NbCRT3 participate in N-mediated defense against TMV: they are required for the expression of a receptor-like kinase IRK that is essential for N-dependent HR [126]. Chen et al. (2005) [66] isolated calreticulin from *N. tabacum* as a TMV MP partner using MP as a bait. Further, these authors demonstrated that *A. thaliana* CRT2 co-localizes with MP at PD, AtCRT1a interacts with MP in Y2H system, and *Zea mays* CRT1 binds MP in blot overlay assay. Finally, it was shown that in *N. benthamiana* transgenic plants expressing *ZmCRT1*, both local and systemic transport of TMV was impeded. It was suggested that CRT prevents MP trafficking to PD as in *ZmCRT1*-transgenic plants MP was distributed mainly along microtubules [66]. Therefore, ZmCRT1 was shown to play an antiviral role, inhibiting MP and TMV intercellular transport. In line with these results, NbCRTs were reported to be involved in HR development, thus CRTs could be regarded as antiviral cellular factors.

## 6. Kinases

Some viral movement proteins functionally could be classified as non-cell autonomous proteins (NCAP), i.e., proteins that are synthesized in one cell and function in another [36,138,139]. Among cellular NCAPs, there are transcription factors and other regulatory proteins [140]. The plant cell harbors specific protein kinases that participate in the control of intercellular transport of both endogenic and viral NCAPs [139,141]. It is known that TMV MP undergoes phosphorylation at several Ser/Thr residues in the C-terminal region [67,70,142]. Initially, it was demonstrated that purified MP could be phosphorylated by kinase(s) contained in the plant cell wall fraction at Ser258, Thr261, and Ser265 residues [142]. Later, this list was supplemented with Thr104 phosphorylated by ER-associated kinases [70]. One of the cell wall kinases designated as plasmodesmata-associated protein kinase (PAPK) was isolated from the plasmodesmata-enriched cell wall protein fraction from tobacco suspension-cultured cells using TMV MP as a bait and demonstrated to co-localize with MP in vivo [68]. Moreover, PAPK phosphorylates both TMV MP and some cellular NCAPs in vitro [68].

Phosphorylation of TMV MP plays a dual role in the functioning of this protein [143]. First, MP phosphorylation may control additional events in the viral life cycle, such as translation/replication of viral RNA: TMV RNA in complex with MP had been neither translatable nor replicatable until MP became phosphorylated by cell wall enriched fractions from *N. tabacum*, as was shown in vitro and in isolated plant protoplasts [144]. Second, MP phosphorylation affects its ability to mediate intercellular transport and move to neighboring cells [67,69].

The important role of phosphorylation in the regulation of TMV MP transport was demonstrated by Waigmann et al. [67]. It was shown that mutant forms of TMV containing substitutions (Ser258, Thr261, and Ser265 were replaced by negatively charged amino acids) that mimic phosphorylation of specific Ser/Thr residues in the MP C-terminal part can neither infect *N. tabacum* nor move from cell to cell in these plants. The same effect was demonstrated for a TMV mutant containing Asp instead of Thr104 in MP. Notably, the replacement of Thr104 with neutral Ala did not affect TMV infection [70]. However, such phosphorylation-dependent regulation appeared to be species-specific: for other members of the *Nicotiana* genus—*N. clevelandii*, *N. benthamiana*, *N. glutinosa*—the effect of MP phosphorylation on infection development was different [67,69]. It was demonstrated that selective mimicking of phosphorylation at one site enhances MP cell-to-cell transport, whereas mimicking of phosphorylation at two or three sites results in the suppression of TMV spread. Carboxyterminal phosphorylation is not required for cell-to-cell movement of TMV MP in *N. tabacum*, *N. benthamiana*, and *N. clevelandii*. In contrast, the phosphorylation of MP is required in *N. glutinosa* for efficient movement. Phosphorylation-mimicking mutant was shown to be transport competent in *N. benthamiana, N. clevelandii*, and *N. glutinosa*, but is severely impaired in movement in the *N. tabacum* host, indicating that in this host phosphorylation negatively regulates the MP capacity to move itself between cells [67,69]. It has also been demonstrated that MP’s ability to perform intercellular transport determined by phosphorylation is closely related to the intracellular localization pattern of the protein, leading to speculation that phosphorylation affects both MP cell-to-cell movement and its PD targeting [69]. These studies support the hypothesis that for TMV/*N. tabacum*, virus–host pair carboxyterminal MP phosphorylation is beneficial: it serves a means for the prevention of severe negative effects for the host plant and, on the other hand, it allows the virus to switch from the “promotion” of local transport to replication and reproduction at the late stages of infection. However, this mechanism is likely functional only in *N. tabacum*, because, in contrast, *N. glutinosa* supports effective intercellular transport of phosphorylated MP. Therefore, in different modes of host–virus interactions, even for closely related plant species, distinct ways of adaptation could be discovered, because the phosphorylation-mediated inactivation mechanism of MP transport function seems to be limited to *N. tabacum* among the panel of other tested hosts from the *Nicotiana* genus.

## 7. Pectin Methylesterases

Pectin methylesterases (PMEs) are cell wall enzymes that play a key role in cell wall remodeling during plant growth and development [145]. PMEs remove methyl groups from pectin, leading to the formation of methanol [146], which acts as a signal molecule during plant–pathogen interactions [147]. *N. tabacum* PME was demonstrated to bind TMV and TVCV MP in vitro by blot overlay assay and affinity chromatography with MP as a bait [71,72]. The same approach was applied to confirm TVCV MP binding to *A. thaliana* PME [74]. TMV MP/PME (*Solanum lycopersicum*) interaction was also confirmed in the Y2H system [72]. Moreover, indirect evidence of PME participation in the development of TMV and TVCV infection was obtained in studies where the PME inhibitor (PMEI) was overexpressed and thus PME activity downregulated: TMV local spread in transgenic *N. tabacum* plants expressing *PMEI* from kiwi was reduced significantly compared to wild-type plants; moreover, systemic infection and symptoms development were delayed. Similar results were obtained for TVCV infection in transgenic *A. thaliana* plants with additional *PMEI* (AtPMEI-2) copy [74]. However, although the MP domain responsible for MP/PME interaction was identified [72], it is still not clear where exactly this interaction occurs in the cell and the mechanism through which PME affects TMV/TVCV intercellular transport [148].

## 8. Non-Cell-Autonomous Pathway Protein (NCAPP)

TMV MP is able to move from cell to cell independently of viral RNA during infection and likely performs “conditioning” of the cells adjacent to the primary infected one, creating a favorable environment for the viral propagation ahead of infection [36]. Thus, due to this feature, TMV MP could be regarded as NCAP similar to cellular proteins that function beyond the cell in which they were synthesized. The mechanism of NCAP transport through PD is understudied; however, a putative receptor—non-cell-autonomous pathway protein (NtNCAPP)—was identified in *N. tabacum*. NtNCAPP was reported to be essential for the TMV MP-mediated increase of PD permeability [149]. The direct binding of TMV MP to *N. benthamiana* NCAPP (also known as aldose 1-epimerase-like protein, NbAELP) was demonstrated using blot overlay assay [76]. *NbNCAPP/AELP* was reported to be involved in plant defense reactions induced by gaseous methanol as one of the methanol-activated genes. *NbNCAPP/AELP* overexpression stimulated TMV-GFP reproduction and was shown to facilitate cell-to-cell movement of macromolecules [75]. However, stably transformed *N. benthamiana* plants with downregulated *NbNCAPP/AELP* appeared to support more effective TMV infection compared to the wild-type plants. In addition, a negative correlation between *NbNCAPP/AELP* and *PME* levels was revealed. Therefore, the following model was proposed: PME activation leads to methanol emission that induces *NbNCAPP/AELP* expression; these events result in the facilitation of viral intercellular transport due to the general methanol effect and, in particular, to interaction with MP, while at the same time NbNCAPP/AELP downregulates *PME* expression likely via affecting its promoter, and competes with PME for secretion and maturation [76]. Taking into account that both PME and NbNCAPP/AELP were shown to be MP-interacting proteins facilitating TMV intercellular transport, it could be assumed that they act at different stages of MP intra- and intercellular movement; on the other hand, each of them might be essential for the viral infection at the particular time point.

## 9. Reversibly Glycosylated Polypeptides

Reversibly glycosylated polypeptides (RGPs) participate in cell wall metabolism during plant growth and development as the majority of these proteins, which belong to class 1, possess mutase activity and are able to catalyze the conversion of UDP-arapiranose to UDP-arabinofuranose [150]. In addition to this important function, RGPs have been demonstrated to be involved in response to viral infection [77,78,151,152]. Moreover, *N. benthamiana* RGPs (NbRGPs) were reported to be virus-induced. NbRGP1 was shown to interact with TMV MP in vitro in blot overlay assay and in vivo in the BiFC system [78]. Transient overexpression of either *NbRGP1-3* leads to a decrease in TMV local spread and reproduction [78], while their downregulation by VIGS, on the contrary, results in more efficient cell-to-cell movement of MP tagged with GFP and TMV-GFP systemic transport [77]. Both transient and stable increased expression of *RGPs* correlates with upregulated PD callose accumulation, which allows us to suggest that RGPs affect viral spread and PD permeability via callose-dependent mechanism [78,152,153]. However, taking into account MP/RGP interaction, another mechanism underlying the limiting of viral infection spread could be based on RGP interfering with MP transport function. RGPs have cytoplasmic localization and are associated with Golgi membranes [78,154,155]; however, it should not be excluded that upon viral infection RGPs could be re-localized to PD, where they probably create a physical or functional barrier for viral intercellular transport [78,153].

## 10. Conclusions

The study of the interactions between viruses and plants has a very long history. Our knowledge of the viral and cellular factors that participate in these interactions and define the route of infection development widens with every new discovered component and partnership. However, each novel result that resolves some questions gives rise to new ones. Many aspects of plant–virus interactions, even for the most studied TMV, are still to be elucidated. In general, the full dynamic picture of these interactions, at all stages of viral pathogenesis, starting from the events in the primary infected cell and ending with the development of systemic infection, hardly exists and thus remains to be created.

**Figure 2:**
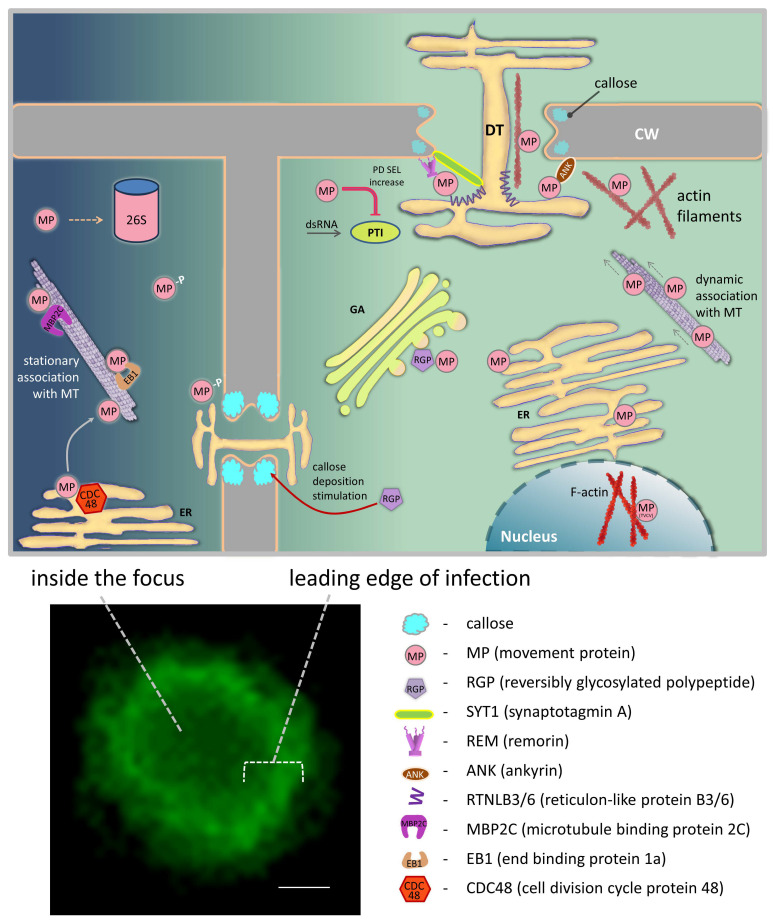
Schematic representation of MP interactions with the cellular partners at the leading edge of infection and behind it, inside the focus. Infection focus on an *N. benthamiana* leaf infected with TMV-GFP vector containing GFP instead of CP, bar = 200 µm. 26S—26S proteasome; CW—cell wall; DT—desmotubule; ER—endoplasmic reticulum; GA—Golgi apparatus; MT—microtubules; PTI—PAMP-triggered immunity.

Here, we have summarized data on TMV MP cellular partners that were identified using various approaches (Table 1, Figure 2).

On the one hand, the localization of these interactions is an important issue to be addressed in many cases, as some potential partners seem to be spatially separated from MP (for example, proteins of ER lumen or cell wall residents). On the other hand, there are numerous examples of redistribution of cellular proteins upon stress or viral infection, thus none of the potential partners should be excluded from the list until a thorough investigation has been conducted. Moreover, the time point, i.e., the stage of viral infection, introduces additional parameters to be taken into account, because the localization of MP and cellular factors varies depending on the stage of infection.

The question of protective reaction induction and/or suppression mechanisms that make it possible to achieve a balance between efficient viral propagation and successful plant growth and reproduction is no less intriguing. Productive viral infection, but limited by plant defense reactions to the particular extent, is likely to be advantageous both for the virus and the plant [156]. To reach such an equilibrium, both pro- and antiviral cellular factors are essential.

## Figures and Tables

**Table 1:** Cellular partners of tobamoviral MPs.

Partner	Virus	Identification Method, Plant	Description	Effect on Viral Infection Development (Functional Tests)	Pro- or Anti-	Reference
Microtubules (tubulin)	TMV	Pull-down assay; in vitro MP/tubulin or MP/microtubule binding; in vivo co-localization studies; BY-2 cells and protoplasts infected with TMV or TMV-GFP	Component of the cytoskeleton	Tobacco mutants with defective microtubule dynamics show reduced susceptibility to TMV infection; on the other hand, disrupting microtubules with drugs or inducing tubulin gene silencing did not affect TMV intercellular movement	Pro-viral: microtubules are essential for TMV local spread and the formation of the viral factories, but their role in mediating MP trafficking to PD is debated	[44,45,46,47,48,49,50]
Myosins VIII-1, VIII-2 and VIII-B	TMV	The dominant negative inhibition (overproduction of myosin tails); transient expression of the myosins tails in inoculated with TMV-GFP *N. benthamiana* plants; and transient expression of the myosin tails in inoculated with TMV-GFP-JL24 *N. benthamiana* plants	Microfilament-associated molecular motors; myosins participate in macromolecular and vesicular intracellular transport	The mean area of TMV-GFP infection sites was reduced by ∼30–75% if VIII-1, VIII-2, VIII-B, XI-K, and XI-2 myosin tails (that act as a dominant negative mutant in the system) were overproduced; inactivation of myosin VIII-1, VIII-2 or VIII-B disrupts the PD localization of MP	Pro-viral: class XI myosins contribute to the viral propagation and intracellular trafficking, whereas myosins VIII are specifically required for the MP targeting to and virus movement through the PD	[51]
Myosins XI-2 and XI-K	TMV	The dominant negative inhibition, which is achieved by overexpression of the myosin tails encompassing dimerization and cargo-binding domains; fluorescence recovery after photobleaching (FRAP) assay	Microfilament-associated molecular motors; myosins participate in the macromolecular and vesicular intracellular transport	Inhibition of myosins XI-2 and XI-K affects the subcellular localization of MP and the structure and dynamic behavior of the ER network. Overexpression of myosin XI-K tail inhibits the systemic movement of TMV	Ambiguous: expression of myosin XI-K tail resulted in the abolishment of the systemic spread of TMV-GFP; virus-induced gene silencing of the *N. benthamiana* myosin XI-2 gene, but not three other myosins, inhibited TMV movement	[51,52]
F-actin	TMV	Pull-down assay with actin monomers and polymeric actin, co-localization with F-actin; *N. benthamiana*	A component of cytoskeleton	TMV MP has an F-actin severing activity in vitro	Pro-viral: inhibition of F-actin depolymerization blocks the increase of PD SEL induced by TMV MP	[44,53]
F-actin-containing filaments in the nucleus	TVCV (but not TMV)	Fluorescence microscopy: analysis of GFP-tagged TVCV MP intracellular distribution and co-localization studies with nuclear proteins in *N. benthamiana* and *A. thaliana*	Nuclear F-actin filaments are associated with chromatin, involved in nuclear dynamics and gene expression regulation	TVCV with mutant MP variant lacking nuclear localization signal (NLS) does not support effective infection: attenuated disease symptoms and delayed systemic infection are observed	Pro-viral: MP interaction with nuclear F-actin filaments was shown to be necessary for efficient TVCV local spread and systemic infection	[42,43]
MPB2C	TMV	Yeast SRS system for detection of protein interaction at the plasma membrane; *Nicotiana tabacum*; confirmed by overlay assay and co-localization studies of proteins with fluorescent tags in *N. benthamiana*	MPB2C is a microtubule-associated protein, with structural properties similar to the myosin/kinesin super family. It withdraws MP and “holds” it in association with microtubules, reducing MP concentration at PD	Transient overexpression or transgenic expression resulted in decreased intercellular transport and increased resistance to tobamoviral infection; *MPB2C* virus-induced gene silencing (VIGS) did not lead to impaired intercellular transport of MP or TMV spread. However, MP association with microtubules was negatively affected	Ambiguous: overexpression suppresses tobamoviral infection while knockdown does not affect it, thus MPB2C possibly participates in the switching between intercellular spread and reproduction at the late stages of infection	[54,55]
EB1a	TMV	Pull-down assay of BY-2 cells proteins with TMV MP as a bait, blot overlay	Microtubule end-binding protein 1a (EB1a) of *Arabidopsis thaliana* is a microtubule plus-end-tracking protein that regulates microtubule dynamics and promotes end-on attachment to different cellular sites	*N. benthamiana* leaves were agroinfiltrated first with TMV-MP:RFP and two days later with AtEB1a:GFP: overexpression of *AtEB1a:GFP* inhibits TMV cell-to-cell movement	Antiviral or regulatory: overexpression of *AtEB1a:GFP* significantly reduces the efficiency of viral intercellular transport	[56]
SYT1 (SYTA)	TVCV, TMV	Initially yeast SRS screen: *A. thaliana* proteins, MP of CaLCuV (*Begomovirus*) as a bait. Confirmed for TMV MP: pull-down assay; BiFC; Y2H	SYT1 (SYTA) is a component of ER–PM contact sites; together with SYT5 and SYT7 it participates in the tethering of these membranes and the formation of membrane contact sites (MCSs); a significant amount of cellular SYT1 was shown to be localized near PD	CaLCuV infection is delayed and TMV MP and CaLCuV MP cell-to-cell spread are inhibited in *SYT1* knockdown line. SYT1 dominant-negative form (transient expression in *N. benthamiana*) inhibited cell-to-cell trafficking of TMV MP and CaLCuV MP; PD targeting activity of the TMV PD localization signal was substantially reduced in an *A. thaliana syta* knockdown line	Pro-viral: downregulation suppresses TMV MP PD targeting and intercellular spread	[57,58,59,60]
NbREM1.5	TMV	BiFC	Membrane proteins located in the microdomains; negative regulator of PD permeability	*NbREM1.5* co-expression with TMV-GFP significantly hampered TMV cell-to-cell movement while *NbREM1.5* suppression via VIGS stimulated TMV-GPF intercellular transport	Antiviral: upregulated expression led to a decrease in TMV local spread, downregulated—facilitated; NbREM1.5 stimulates PD callose deposition	[61]
NtREM1.2	Tomato mosaic virus (ToMV)	BiFC	Membrane proteins located in the microdomains; NtREM1.2 PD localization is not confirmed	Transient expression of *NtREM1.2-DsRed* with ToMV-GFP leads to significant stimulation of viral intercellular spread at 72 h after infiltration	Pro-viral: likely stimulates viral intercellular movement	[62]
Reticulon-like proteins RTNLB3 and RTNLB6	TMV	Y2H, FRET-FLIM, co-immunoprecipitation	Membrane proteins that mediate membrane bending and curving, believed to be responsible for desmotubule formation, interact with SYT1 and two remorins—REM1.2 and REM1.3	No data available on RTNLBs’ effect on TMV infection or transport	Unknown	[63]
ANK	TMV	Blot overlay assay, BiFC; *N. tabacum*	Ankyrin-repeats containing proteins participate in multiple processes, such as plant growth and development, hormone response, response to biotic and abiotic stresses	System: transgenic tobacco lines with RNAi for *ANK* or *ANK* superexpression; MP-YFP-encoding construct bombardment and MP-YFP intercellular spread assessment; *ANK* gene suppression resulted in impeded MP-YFP intercellular spread, while *ANK* overexpression led to stimulation of MP-YFP transport	Pro-viral: *ANK* co-expression with *MP* leads to PD callose reduction and enhancement of MP intercellular transport and, consequently, more efficient TMV-DsRed local transport	[64]
NtMPIP1	TMV	Y2H, Y3H; TMV MP as a bait; confirmed by blot overlay assay; *N. tabacum*	NtMPIP1, a member of the type I DnaJ chaperone family	*NtMPIP1* VIGS significantly inhibited TMV spread; in Y3H system, the interaction between NbMPIP1/MP/NTH201 was demonstrated	Pro-viral: NtMPIP1 downregulation by PVX-based VIGS led to reduced local spread of TMV:GFP and less efficient viral RNA accumulation	[65]
CDC48	Oilseed rape mosaic virus (ORMV) and TMV	Tobacco CDC48 co-IP with TMV MP; FRET-FLIM approach for TMV and ORMV MPs; *A. thaliana*, *N. benthamiana*	ER chaperone, which participates in retrotranslocation of proteins from the ER to the cytosol during ER-assisted protein degradation	Overexpression of *CDC48* ahead of infection reduces the size of viral infection sites because of MP relocalization to microtubules, leading to the downregulation of intercellular movement	Ambiguous: exploited by the virus for switching from intercellular movement to replication and virus particles accumulation; CDC48 “extracts” MP from ER inclusions containing viral factories and directs it to microtubules and for degradation at the late stages of infection	[40]
CRT1,2	TMV	Affinity chromatography on immobilized TMV MP column of *N. tabacum* cell wall proteins; interaction confirmed by co-localizaiton studies (FRET) of AtCRT1 and TMV MP; Y2H system	ER-resident chaperones participating in protein folding and Ca^2+^ homeostasis	Transgenic *N. benthamiana* plants overexpressing *ZmCRT1*; *A. thaliana* plants expressing *AtCRT2:YFP* and *MP:GFP*	Antiviral: interferes with TMV MP intracellular traffic; increased production of CRT leads to MP relocalization to microtubules instead of PD that results in decreased efficiency of TMV intercellular spread	[66]
PAPK (plasmodesmata-associated protein kinase) and putatively other cell wall kinases	TMV	Tobacco BY-2 suspension-cultured cells	Casein-kinase that phosphorylates some cellular NCAPs in vitro as well as MP in Ca^2+^—independent manner	Phosphorylate MP at Ser 258, Thr 261, and Ser 265	Ambiguous: C-terminal MP phosphorylation is beneficial for both TMV and *N. tabacum*: it serves as a means for the prevention of severe negative effects for the *N. tabacum* host plant and allows the virus to switch from “promotion” of local transport to replication and reproduction at the late stages of infection	[67,68,69]
ER-associated kinase(s)	TMV	In vitro MP phosphorylation in the presence of [γ-^32^P]ATP with microsomal fractions obtained from *N. tabacum* (cv. Samsun) leaves	ER-associated kinases perform protein phosphorylation	Phosphorylates MP at Thr104; TMV substitution mutants with Asp (mimicking phosphorylation) instead of Thr104 resulted in much smaller local lesion development in *N. tabacum* cv. Xanthi plants than Thr104-containing TMV or TMV with Thr104-to-Ala substitution; MP phosphorylation at Thr104 leads to the impediment of TMV intercellular spread	Ambiguous: replacement of potentially phosphorylated Thr104 with neutral Ala does not lead to any adverse effects for viral infection development, while phosphorylation mimicking mutation results in transport suppression; thus, it could be the mechanism of virus infection restriction for reduction of negative effects to the host and at the same time might be exploited by the virus to transiently modulate MP functions	[70]
PME	TMV, TVCV, CaMV	Identification by blot overlay assay: *N. tabacum* cell wall proteins are on the membrane, MP is in the solution; purification by affinity chromatography using MP as a bait; confirmed in Y2H; TVCV MP-based affinity chromatography revealed its binding with *A. thaliana* PME	Cell-wall proteins that perform pectin de-esterification; participate in cell wall remodeling	Deletion of PME-binding domain from MP led to impairment of viral intercellular movement; TMV systemic movement was suppressed in transgenic tobacco expressing *PME* coding sequence in antisense orientation	Pro-viral, interaction between MP and PME is essential for viral cell-to-cell movement	[71,72,73,74]
NCAPP/AELP	TMV	Blot overlay assay	Positive PD regulator; induced in response to TMV infection and gaseous methanol treatment	Co-expression with TMV:GFP stimulates reproduction of viral vector and its intercellular transport	Pro-viral: increased expression stimulates TMV intercellular movement; however, transgenic *N. benthamiana* plants with downregulated expression were more susceptible to TMV infection than wild-type plants	[75,76]
NbRGP1	TMV	Blot overlay assay, BiFC; *N. benthamiana*	RGPs play an important role in L-arabinose metabolism supplying the cell with UDP-arabinofuranose for cell wall and glycoprotein biosynthesis; in addition, RGPs participate in plant–virus interactions and are regarded as negative PD regulators	*NbRGP1, 2* transient overexpression leads to reduced TMV:GFP viral vector RNA accumulation and suppressed TMV:GFP local spread; NbRGPs VIGS resulted in more effective TMV:GFP and MP:GFP intercellular transport and accelerated TMV systemic infection	Antiviral: *NbRGPs* upregulated expression limits viral local transport in a callose-dependent mechanism and via direct interaction with MP	[77,78]

BiFC—bimolecular fluorescence complementation; co-IP—co-immunoprecipitation; FRAP—fluorescence recovery after photobleaching; FRET-FLIM—Förster resonance energy transfer (FRET) measured using fluorescence-lifetime imaging microscopy (FLIM); SRS—yeast two-hybrid SOS-recruitment system; VIGS—virus-induced gene silencing; Y2H—yeast two-hybrid system; Y3H—yeast three-hybrid system.

## Data Availability

No new data were created in this study. Data sharing is not applicable to this article.
